# Evaluation of the Influence of Varied Juncao Grass Substrates on Physiological and Enzymatic Reactions of *Pleurotus ostreatus*

**DOI:** 10.3390/cimb46090563

**Published:** 2024-08-28

**Authors:** Irambona Claude, Nsanzinshuti Aimable, Hatungimana Mediatrice, Hengyu Zhou, Dongmei Lin, Penghu Liu, Zhanxi Lin

**Affiliations:** 1 National Engineering Center of JUNCAO Technology, College of Life Science, Fujian Agriculture and Forestry University, Fuzhou 350002, China; claudebiofafu@gmail.com (I.C.); nsanziaima@gmail.com (N.A.); mediatunga@gmail.com (H.M.); ricardozhou@foxmail.com (H.Z.); phliu1982@163.com (P.L.); 2 Rwanda Agriculture and Animal Resources Development Board, Huye P.O. Box 5016, Rwanda

**Keywords:** substrates, *Pleurotus ostreatus*, biological efficiency, yield, enzymes

## Abstract

*Pleurotus ostreutus* is one of the world’s most commonly consumed mushrooms. The cultivation of mushrooms using wood resources usually results in environmental issues such as deforestation. Juncao grasses, namely (JJ) *Cenchrus fungigraminus*, (AR) *Saccharum arundinaceum*, and (MS) *Miscanthus floridulus*, supplemented with 20% wheat brain, 1% ground coffee, 1% gysum, and 1% lime, were used as the culture mediums in this research, which offers a composting system with a simple formulation that is cheap and feasible for small farms to use in cultivating oyster mushrooms. The present study assessed the different juncao grasses as substrates for growing *Pleurotus ostreatus* given their enzyme activities, growth, and yields. The results demonstrated that the yields of *pleurotus ostreatus* grown on JJ, AR, and MS substrates were significantly different at the level of 0.05 and were recorded as follows: 159.2 g/bag, 132 g/bag, and 65.1 g/bag on average, respectively. The biological efficiency of *Pleurotus ostreatus* cultivated in three different substrates was 75.2%, 63.4%, and 28.7%, respectively. Lignin peroxidase (LiP) was the most active enzyme in each culture material among the other enzyme activities expressed differently between the substrate and growing stages. At the same time, other enzyme activities were differently expressed between the substrate and different developmental stages. Nutrient analysis revealed significant variations, with differences in polysaccharides, proteins, and amino acids among substrates, as well as the presence of heavy metals such as arsenic, lead, mercury, and cadmium in all samples within safe limits. The obtained results indicated that *Saccharum arundinaceum* is a good substrate in place of *Cenchrus fungigraminus*, and that using *Miscanthus floridulus* is not productive. Moreover, the juncao grasses offer a sustainable approach that reduces reliance on wood-based substrates and enhances environmental sustainability.

## 1. Introduction

Culturing edible mushrooms is an important worldwide commercial activity. The oyster mushrooms of the genus *Pleurotus* are the world’s third-largest commercially cultivated mushroom. They can utilize a wide range of available culture materials due to their great adaptability [1,2]. The mushroom fruit body is low in calories, fat, and cholesterol, while rich in protein, carbohydrates, fibers, vitamins, and minerals. These nutritional properties make mushrooms a very beneficial dietary food [3]. *Pleurotus* mushroom cultivation is an economically viable and eco-friendly process for converting various agro wastes into human food. *Pleurotus* mushrooms are cultivated on a large scale globally, accounting for 27% of global mushroom production [4,5].

In Asia, the *pleurotus* mushroom industry has increased rapidly due to low production costs and high-yielding capacity. The cultivation processes include substrate processing, casing, and temperature shocks [6].

The culture materials, also called substrates, are the most significant factors in the quality and yield of edible fungi and medical mushrooms [7,8]. Substrates containing cellulose, hemicelluloses, and lignins are sources of carbon, nitrogen, and other essential minerals. In general, mushrooms can decompose organic matter, particularly cellulose, hemicellulose, and cellulose, from which they produce a series of extracellular enzyme activities that were tested [9]. The quality of protein production and flavor formation depends on the substrates and also offers the nutrition, moisture, and energy that mushrooms require to grow and fruit [10].

The correct concentrations of nitrogen and carbohydrate sources are required to achieve a high mushroom yield in terms of dry weight. High glucose concentrations can affect yield and inhibit the growth of many mushrooms. Environmental factors such as humidity, temperature, carbon dioxide, and oxygen are very essential and affect the growth and production yield of mushrooms [11,12].

Cultivation of mushrooms using wood resources usually results in environmental issues of deforestation. Little research has reported on using Juncao grasses as the solution to mushroom growing. Therefore, three different juncao grasses, *Cenchrus fungigraminus*, (AR) *Saccharum arundinaceum,* and (MS) *Miscanthus floridulus,* from the National Juncao Engineering Research Center were used in this study to grow *Pleurotus ostreatus* [13].

## 2. Materials and Methods

### 2.1. Spawn Preparation and Mushroom Material

The *Pleurotus ostreatus* (P969) strain was obtained from the National Juncao Engineering Research Center of Juncao Technology in Fuzhou City, Fujian Province. As is demonstrated in Supplementary Figure S1, the strain was grown on Potato Dextrose Agar (PDA) medium (PSA) at 25 °C for regular subculture. The second spawns were prepared in 850 mL polypropylene plastic bags with a total of 500 g of dry matter counted as 78% *Dicranopteris Dicholoma* supplemented with 20% wheat bran, 1% calcium carbonate, 1% lime in terms of dry weight, and 60~65% water content, and then sterilized at 121 °C for 2 h. After cooling to room temperature, 10 cm of the mycelium of the oyster mushroom s were inoculated into each bag of sterilized substrate. The inoculated substrates were incubated at room temperature to mature into spawn.

### 2.2. Substrate Preparation and Inoculation

Three lignocellulosic substrates, *Cenchrus fungigraminus* (JJ), *Saccharum arundinaceum* (AR), and *Miscanthus floridulus* (MS), were obtained from the National Juncao Engineering Research Center. In Supplementary Figure S2a, it is shown that 77% of each Juncao grass substrate (JJ, AL, and MS) was supplemented with the following materials: 20% wheat brain, 1% coffee grounds, 1% of gypsum, and 1% lime thoroughly mixed with a mixture machine. Water was added to reach a moisture content of approximately 60–65%, where if you hold it tightly, you can see that the water seems to lick between your fingers. As is demonstrated in Supplementary Figure S2b, the substrates were filled in plastic bags, each bag with a volume between 800 g–1 kg, sterilized in an autoclave machine separately at 121 °C for 120 min, and then cooled down for 24 h. After cooling to room temperature, 3 g of mycelium was inoculated into each plastic bag, and twenty-four culture bags were used for each substrate. Inoculated substrates were incubated at room temperature until fully colonized.

### 2.3. Incubation and Harvest

The inoculated substrates were kept in an incubation room at 27 °C and 60~70% relative humidity. After total mycelium colonization, the bags were transferred to a growing room, in which the temperature was maintained at 24 °C, and kept at a relative humidity of about 85–90%, as illustrated in Supplementary Figure S2c. For all substrates, three flushes of mushrooms were harvested from each of the culture bags when the in-rolled margins of the mushroom caps began to flatten, as displayed in Supplementary Figure S2d. The time from inoculation to the first harvest and total harvesting time were recorded. At every flush, the harvested fruiting bodies were weighed, and mushroom size was measured. The length and thickness of the stipe, the diameter of the cap, and the number of effective fruiting bodies per bunch were measured at the first, second, and third flush and the means were also determined [14,15]. As illustrated in the Supplementary Table S1, at the end of the harvest period, the accumulated data were used to calculate the total yield and the BE. The biological efficiency was calculated by the ratio of fresh fruiting body weight (g) per dry weight of substrates (g), expressed as a percentage [16].

### 2.4. Experimental Design and Data Analysis

The following experiments were performed at the National Juncao Engineering Laboratory in Fuzhou. The experiment was laid out in a randomized complete block design with three replications of 24 culture bags each. The data were compared using analysis of variance (ANOVA) and significant differences between means were determined by the honest significant difference (HSD-Tukey test) at the level of *p* < 0.05. Data were reported as mean values ± standard deviation of three independent replicates (*p* < 0.05, 95%) [17].

### 2.5. Analysis of Enzyme Activities

The Elisa method, as proposed by A.T. Hoang [18], was used to assay for the enzyme activity of laccase, manganese peroxidase (MnP), lignin peroxidase, xylanases, exoglucanases (FPase), and endoglucanase in the samples from the mycelium and fruiting stage. Add 10 µL of reconstituted standard mixed with 990 µL of incubation buffer in one well as a standard well of the micro-ELISA test strip plate. Add 40 µL of the sample dilution buffer into the well of the testing sample. Then, add 10 µL of the testing sample to the wells and mix gently, except for the blank well. A total of 100 µL of HRP-conjugate reagent was then added to each well in each plate, closed with an adhesive strip, and incubated for 30 min at 37 °C. Then, further constant numbers of 1000 Titer stock of the washing solution were prepared with distilled water and stored until used. Discard the closed plates and dry them, using a swing to uncover them. Then, add washing buffer again and hold for 30 s then drain. Further incubation was conducted with 50 µL of HRP-conjugate reagent in all but the blank well of the plates at 37 °C, with another washing process for 30 min using the buffer. A total of 50 µL of each chromogenic solution A and chromogenic solution B is added gently to maintain the volume in each well and the plate is incubated at 37 °C for 15 min under light protection. Following the previous report [19], the reaction was next terminated by the addition of 50 µL of H_2_SO_4_ solution into each well. Once the solution has changed color from blue to yellow, the absorbance (optical density—OD) was read within the first 15 min of adding the stop solution at a wavelength of 450 nm. Based on the standard curve prepared, the sample density was calculated from the OD value. For each enzyme, the actual sample density was the dilution factor multiplied by the sample density on the graph.

## 3. Results

### 3.1. Effect of Different Substrates on Mycelium Running and Biological Efficiency

The results showed (Table 1) that the mycelium running in three different substrates was between 28 days and 40 days. The substrate named *Cenchrus fungigraminus* (JJ) had rapid mycelium growth for 28 days, followed by *Saccharum arundinaceum* (AR) with 32 days and *Miscanthus floridulus* (MS) with 40 days. Previously, King T.R. [20] reported quite similar results to those found in this research. Therefore, there is no significant difference in the yield, stipe, cap, and BE of *pleurotus ostreatus* planted in JJ and AR. The *Miscanthus floridulus* (MS) showed a high contamination rate (65%) during the incubation period, which resulted in the low yield, cap, stipe, and mycelium running of *Pleurotus ostreatus.*

### 3.2. Effect of Different Substrates on the Nutritional Composition of the Fruiting Body

Fundamental food characteristics, such as the polysaccharide, fiber, protein, and carbohydrate content of oyster mushrooms grown on different substrates, are presented in Table 2. The effects of substrates JJ and MS were not significant on the polysaccharide content (2.31–2.43%) of cultivated mushrooms, while polysaccharide content (2.77%) in the AR substrate was significantly higher than JJ and MS. The result was quite similar to the value stated by Wang et al. [17,21]. The study indicated that the fruiting bodies of oyster mushrooms PO grown on all substrate formulas are quite rich in fiber, carbohydrates, protein, amino acids, and antioxidants, making them excellent foods that can be used in low-calorie diets.

The protein level of the mushrooms was significantly different in all substrates. The highest protein content was found in JJ, followed by AR and MS, at 1.84%, 1.48%, and 1.39%, respectively. The differences in the protein content of mushrooms grown on different substrates could be due to the varying nitrogen content of substrates. The high nitrogen content of JJ and AR contributed to the higher protein content of fruiting bodies, while MS provided less nitrogen, which could be attributed to the nitrogen utilization efficiency of the species. The fiber content of oyster mushrooms on a dry weight basis was not significantly different in all substrates. The amount of carbohydrates was not significant in MS and JJ at 67.93% and 64.80%, respectively, and was significantly different from AR at 60.63%, which is recommended for cultivation as a second option. In this study, the carbohydrate content is higher than that reported by H. T. Hao et al., and the total carbohydrate content in this study was higher than the reported 40.64~55.92% grown in different substrates. The free amino acids of *Pleurotus ostreatus* were significantly different (*p* ≤ 0.05) in all substrates. The highest free amino acid content was found in JJ, followed by AR and (MS), at 0.39%, 0.29%, and 0.27%, respectively.

The fruit body of *Pleurotus ostreatus* harvested in the substrates JJ and MS showed high antioxidant activity (16.86%, 16.70%), respectively, but no significant difference compared to the content in AR (13.83%), and the results were significantly different at the level of *p* < 0.05. The results shown had similarities to the results reported by P. Diamantopoulou et al. [17], where the antioxidant activity was influenced by the substrate.

### 3.3. Concentration of Heavy Metals

The heavy metals (Cadmium, Arsenic, Plumbum, and, Mercury) were determined in the fruiting body of *Pleurotus ostreatus*. Previous research has indicated that edible fungi accumulate different concentration levels of heavy metals [22]. As indicated in Table 3 below, the content of four heavy metals was tested from the fruiting body of pleurotus ostreatus grown in three different mushroom substrates.

The concentrations of Plumbum (Pb), Mercury (Hg), and Arsenic (As) in the fruiting body of *Pleurotus* grown in the substrates JJ, MS, and AR were significantly different at the level of 0.005, and the Cadmium concentration in JJ (0.032 mg·kg^−1^) was higher than in MS and AR. The concentration level of mercury (Hg) in the fruiting body of *Pleurotus ostreatus* grown in AR (0.079 mg·kg^−1^) was higher than in MS, followed by JJ, and 0.055 and 0.047 mg.kg^−1^, respectively.

### 3.4. Effect of Enzyme Activity Assay

**Figure 1:**
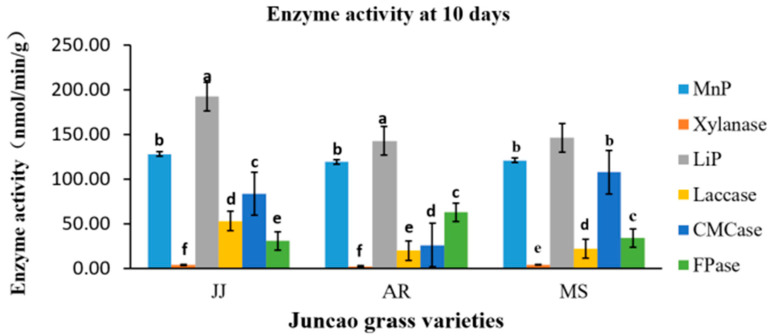
Effect of different substrates on Pleurotus ostreatus enzyme activity on the tenth day. Different letters show significant differences at a *p*-value of 0.05 (*p* ≤ 0.05). LiP: Lignin peroxidase; Laccase; MnP: Manganese peroxidase; Xylanase; FPase: Exo-glucanase; CMCase: Endo-glucanase. Substrates: JJ: *Cenchrus fungigraminus*, AR: *Saccharum arundinaceum*, MS: *Miscanthus floridulus.*

**Figure 2:**
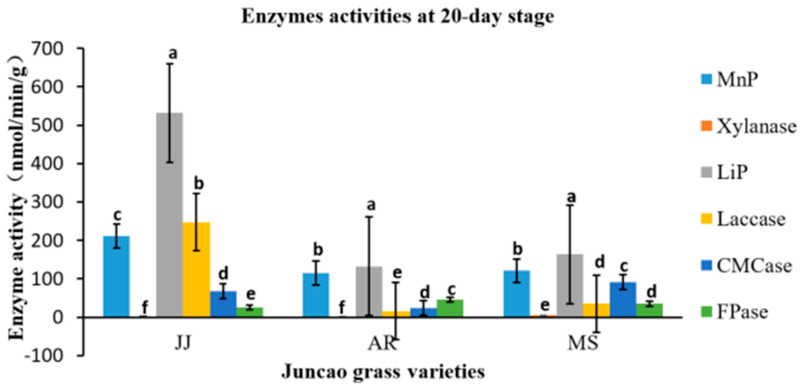
Effect of different substrates on *Pleurotus ostreatus* enzyme activity on the twentieth day. Different letters show significant differences at a *p*-value of 0.05 (*p* ≤ 0.05). MnP: Lignin peroxidase; Laccase; MnP: Manganese peroxidase; Xylanase; FPase: Exo-glucanase; CMCase: Endo-glucanase. Substrates: JJ: *Cenchrus fungigraminus*; AR: *Saccharum arundinaceum*; MS: *Miscanthus floridulus.*

**Figure 3:**
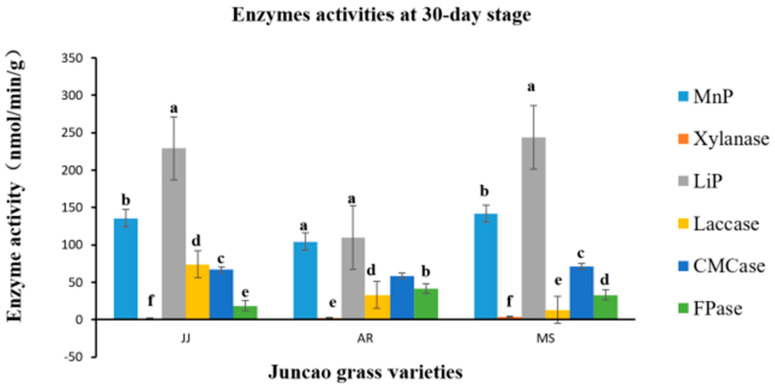
Effect of different substrates on *Pleurotus ostreatus* enzyme activity on the thirtieth day. Different letters show significant differences at a *p*-value of 0.05 (*p* ≤ 0.05). LiP: Lignin peroxidase; Laccase; MnP: Manganese peroxidase; Xylanase; FPase: Exo-glucanase; CMCase: Endo-glucanase. Substrates: JJ: *Cenchrus fungigraminus*, AR: *Saccharum arundinaceum*, MS: *Miscanthus floridulus.*

**Figure 4:**
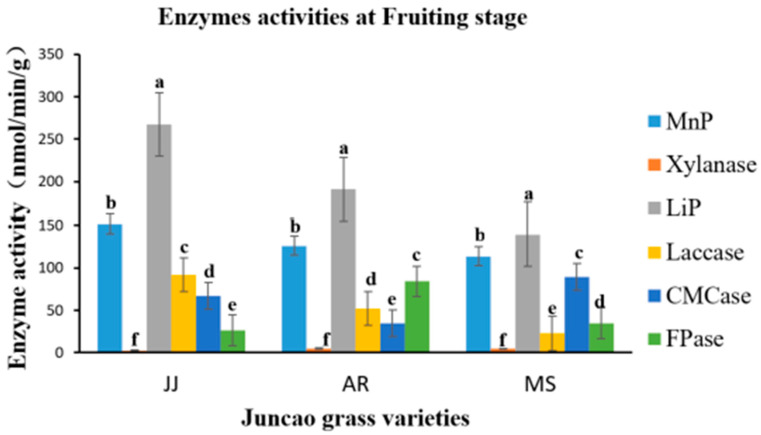
Effect of different substrates on *Pleurotus ostreatus* enzyme activity at the fruiting body stage. Different letters show significant differences at a *p*-value of 0.05 (*p* ≤ 0.05). LiP: Lignin peroxidase; Laccase; MnP: Manganese peroxidase; Xylanase; FPase: Exo-glucanase; CMCase: Endo-glucanase. Substrates: JJ: *Cenchrus fungigraminus*, AR: *Saccharum arundinaceum*, MS: *Miscanthus floridulus*.

The enzyme activity assays were performed on the tenth day after inoculation of *Pleurotus Ostreatus* mycelium growth, up to the fruiting stages. The three substrates had a great participation in all enzyme activity, but their activity differed between developmental stages, as demonstrated in Table 4, Table 5, Table 6 and Table 7 and Figure 1, Figure 2, Figure 3 and Figure 4. Throughout the whole development period, there were significant differences (*p* ≤ 0.05) in the activity of lignin peroxidase in *Pleurotus ostreatus* grown on three substrates. From the tenth day up to the fruiting body stage, the lignin peroxidase (LiP) presented the highest activity in all three juncao grass substrates in different developmental stages, as indicated in Table 4, Table 5, Table 6 and Table 7. At the fruiting body stage, LiP activity in the three substrates JJ, AR, and MS was also high, followed by MnP and Laccase. The observation of the high activity of LiP, MnP, and Laccase in the three different substrates studied in this research is quite similar to the earlier findings of Suryadi and Rameshaih [23,24], who showed that lignin peroxidase is the most efficient and best-known ligninolytic enzyme in fungi.

As illustrated in Table 6, during the 30-day stage, there were substantial differences (*p* ≤ 0.05) in Laccase activity production between the three juncao grass substrates (JJ, AR, and MS). At the fruiting body stage, endo-glucanase and exo-glucanase significantly produced the highest activities in the MS substrate, followed by JJ and AR (Figure 4), and broadly similar findings were reported by Wang. H et al. [25]. The results showed that xylanase activity consistently decreased during all developmental stages of each juncao grass substrate. The results of changes in the activity of six analyzed enzymes in this study are quite similar to those reported by Sitar et al. [26].

## 4. Discussion

*Pleurotus ostreatus*, representing oyster mushrooms, is the most wanted, expansively grown, and consumed macromycete in the whole world. It is also commonly known as common Indian or commercial oyster mushrooms [27]. This study assessed the influence of different substrates on the physiological and biochemical responses of *Pleurotus ostreatus*. Numerous studies have demonstrated that the yield, biological characteristics, nutritional content, and enzyme activities of *Pleurotus Ostreatus* are heavily influenced by the choice of cultivation material.

The utilization of three Juncao grasses as experimental materials for cultivating *Pleurotus ostreatus* is very helpful. Previous research reported by Hu. Y. et al. (2019) demonstrated that using Juncao grass (*M. floridulus*) and cotton seed waste materials can increase the yield and biological efficiency of cultivating *Pleurotus ostreatus*. In this study, the results demonstrated that the substrate with *Cenchrus fungigraminus* had the most rapid mycelia running rate of *Pleurotus ostreatus* (28 days), the highest yield (159 g/bag), and the highest biological efficiency of 75%. This was followed by *Saccharum arundinaceum* with 32 days, 132 g/bag, and 65% BE. These findings were similar to those reported by Hu Yingping et al. [28].

The six enzyme activity assays were performed on the tenth day after the inoculation of *Pleurotus Ostreatus* mycelium growth until the fruiting stages. All three substrates had a significant impact on enzyme activity, but the activity varied between different developmental stages. *Pleurotus ostreatus* showed notable differences in lignin peroxidase activity amongst the three substrates during the culture process, indicating significant differences (*p* ≤ 0.05) with *Saccharum arundinaceum* (AR) and *Miscanthus floridulus* (MS). These findings were consistent with the research conducted by Fernández-Fueyo et al. [29], which highlighted the ability of mycelium growth to decompose lignocellulosic materials.

The nutrient content of polysaccharides, fiber, protein, carbohydrates, antioxidants, and amino acids differed in oyster mushrooms grown on different substrates. The results indicated that substrates JJ and MS did not significantly affect the polysaccharide content (2.31–2.43%) of the cultivated mushrooms. However, the polysaccharide content (2.77%) in the AR substrate was significantly higher than in JJ and MS. This finding was consistent with the research conducted by Diamantopoulou P. et al. [17], which focused on the nutrient compositions of edible mushrooms. Additionally, the protein content of the fruiting body of *Pleurotus ostreatus* cultivated using JJ had the highest protein content of 1.80%. Following that, AR and MS had protein contents of 1.48% and 1.39% respectively. The crude fiber content of P.O in all substrates was 0.49%, 0.50%, and 0.50%, showing no significant differences. In terms of polysaccharide content, *Pleurotus ostreatus* grown in the AR substrate had a higher content of 2.77% compared to JJ and MS. The mean difference was significant at the 0.05 interval in JJ and AR substrates. Nagulwar, M. [30] reported that mushrooms are considered low-calorie food with high nutritional value, containing good-quality proteins and vitamins, particularly the vitamin B complex. The results were closely similar to our findings. The concentration of heavy metals (Cadmium, Arsenic, Plumbum, and Mercury) was determined in the fruiting body of *Pleurotus ostreatus* cultivated in different substrates, namely JJ, AR, and MS. Therefore, the results demonstrated that the concentrations of heavy metals found in the fruiting bodies of *Pleurotus* grown in different substrates were below the minimum allowable levels for edible vegetables as reported in international food regulations, which are similar to the findings of research by Wang et al. [31].

In conclusion, the goal of the present study was to cultivate *Pleurotus ostreatus* on three different culture materials supplemented with wheat straw. However, the analysis of yield and BE was high in the JJ substrate, which was the best culture material, followed by AR. This study also showed a dynamic change in extracellular enzyme activity during mycelia-fulfilled development, the formation of primordia, and the formation of fruiting body stages in different substrates. For this reason, our study shows that changes occur in lignocellulose, where cellulose, lignin, and hemicellulose levels are lower than those in the fresh substrate. 

## Figures and Tables

**Table 1:** Effect of different substrates on mycelium running and biological efficiency of *Pleurotus. ostreatus.*

Substrate	Mycelium Running (Days)	Yield (g)	Stipe Length (cm)	Cap Diameter (cm)	BE Efficiency (%)
JJ	28.7 ± 2.08 a	159.2 ± 11.4 b	7.1 ± 1.4 b	8.33 ± 1.05 b	75.2 ± 5.39 b
AR	32. ± 2.05 b	132.2 ± 9.17 b	6.7 ± 1.32 b	7.30 ± 0.45 b	63.40 ± 4.05 b
MS	40.43 ± 3.07 b	65.10 ± 0.4 a	4.3 ± 0.9 a	4.50 ± 0.79 a	28.7 ± 2.05 a

Different letters in the same column show significant differences at a *p*-value of 0.05 (*p* ≤ 0.05); ±SD at 5% probability; the total number of replicate bags per substrate was 24 bags according to Duncan’s multiple range test. BE: Biological Efficiency; Substrates: JJ: *Cenchrus fungigraminus*, AR: *Saccharum arundinaceum*, and MS: *Miscanthus floridulus.*

**Table 2:** The nutritional content of *Pleurotus ostreatus* cultivated in different substrates.

Substrate	Polysaccharide	Fiber	Carbohydrate	Protein	Amino Acids	Antioxidant
GG	2.31 ± 0.04 a	0.49 ± 0.03 a	64.80 ± 1.37 b	1.84 ± 0.05 c	0.39 ± 0.09 c	16.86 ± 0.38 b
AR	2.77 ± 0.05 b	0.50 ± 0.07 a	60.63 ± 2.32 a	1.48 ± 0.01 b	0.29 ± 0.05 b	13.83 ± 0.12 a
MS	2.43 ± 0.07 a	0.50 ± 0.08 a	67.93 ± 1.42 b	1.39 ± 0.01 a	0.27 ± 0.04 a	16.70 ± 0.30 b

Different letters in the same column show significant differences at a *p*-value of 0.05 (*p* ≤ 0.05) according to Duncan’s multiple range test. Substrates: JJ: *Cenchrus fungigraminus*, AR: *Saccharum arundinaceum*, MS: *Miscanthus floridulus.*

**Table 3:** Comparative analysis of Heavy Metal Concentrations (mg·kg^−1^) in fruiting bodies of *Pleurotus* cultivated across three substrates.

Substrate	Plumbum	Cadmium	Mercury	Arsenic
	(Pb)	(Cd)	(Hg)	(As)
JJ	0.075 a	0.032 b	0.047 a	0.049 c
AR	0.023 b	0.023 a	0.079 c	0.039 b
MS	0.099 c	0.023 a	0.055 b	0.016 a
IFSS	0.9	0.24	0.2	0.3

Different letters in the same column indicate that the heavy metal contents in *Pleurotus* differ significantly from one another at the *p*-value of 0.05. IFSS: International Food Safety Standard.

**Table 4:** Enzyme activities (nmol/min/g) at 10 days in three different juncao grass varieties.

Juncao Grass Variety	MnP	Xylanase	LiP	Laccase	CMCase	FPase
JJ	127.97	3.83	**192.39**	52.97	83.67	30.67
AR	119.14	2.28	**142.7**	19.79	26.07	62.72
MS	120.93	4.09	**146.26**	22.04	107.61	33.84

**Table 5:** Enzyme activities (nmol/min/g) during a 20-day stage in three different juncao grass varieties.

Juncao Grass Variety	MnP	Xylanase	LiP	Laccase	CMCase	FPase
JJ	211.13	2.57	**532.23**	247.38	67.37	25.64
AR	114.82	1.47	**132.31**	15.84	24.03	46.27
MS	121.1	4.47	**163.77**	35.29	90.88	34.86

**Table 6:** Enzyme activities (nmol/min/g) during a 30-day stage in three different juncao grass varieties.

Juncao Grass	MnP	Xylanase	LiP	Laccase	CMCase	FPase
JJ	135.75	0.231	**228.93**	73.84	66.77	18.5
AR	104.45	1.88	**243.82**	12.87	25.3	41.46
MS	141.87	4.05	**109.64**	80.95	71.18	33

**Table 7:** Enzyme activities (nmol/min/g) at the fruiting body stage in three different juncao grass varieties.

Juncao Grass Variety	MnP	Xylanase	LiP	Laccase	CMCase	FPase
JJ	151.35	2.56	**267.19**	91.84	67.3	26.48
AR	126.06	4.72	**191.51**	52.05	34.83	84.12
MS	113.45	4.46	**139.33**	22.83	89.13	34.68

## Data Availability

The data supporting the findings of this study are available on request from the corresponding author.

## Supplementary Materials

The supplementary materials for this article can be found online at https://www.mdpi.com/article/10.3390/cimb46090563/s1, Figure S1: Medium culture and preparation of *Pleurutus ostreatus* spawns in PDA. (A) *Pleurotus* strain inoculated in Petri dishes. (B) Mycelium running in the Petri dishes. Figure S2. Different stages of oyster mushroom cultivation (*Pleurotus ostreatus*) p969. (a) Art of substrate preparation. (b) Sterilized substrate ready for injection. (c) Colonized substrate. (d) First flush for three different grasses. Table S1. Different substrates on mycelium running rate, and biological efficiency of *Pleurotus. Ostreatus.* Table S2. Different enzyme activities (1–4) of *P. ostreatus* grown in varied developmental stages on *Cenchrus fungigraminus* (JJ), *Saccharum arundinaceum* (AR), and *Miscanthus floridulus* (MS supplemented with wheat bran and lime).
