# Supplementary material for: Evaluation of the Influence of Varied Juncao Grass Substrates on Physiological and Enzymatic Reactions of Pleurotus ostreatus
Source: Curr Issues Mol Biol. 2024 Aug 28;46(9):9493–502. doi: 10.3390/cimb46090563 (PMC11429835; doi:10.3390/cimb46090563)
Supplement: Supplementary file 1 [file cimb-46-00563-s001.zip › cimb-3103604-supplementary Figures.pdf]

Supplementary Figures

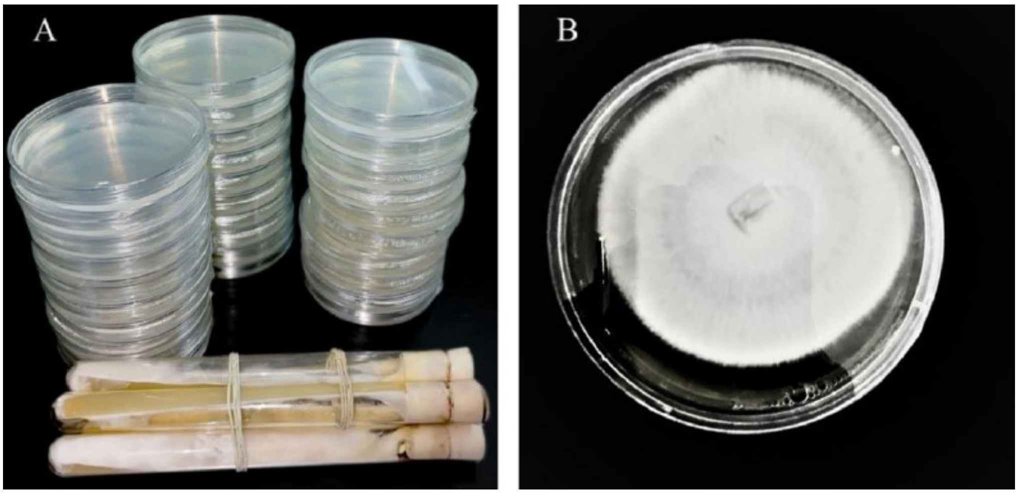

Figure S1. Medium culture Preparation of *Pleurutus ostreatus* spawns in PDA. (**A**) *Pleurotus* strain and inoculated in petric dishes. (**B**) Mycelium running in the petri dishes.

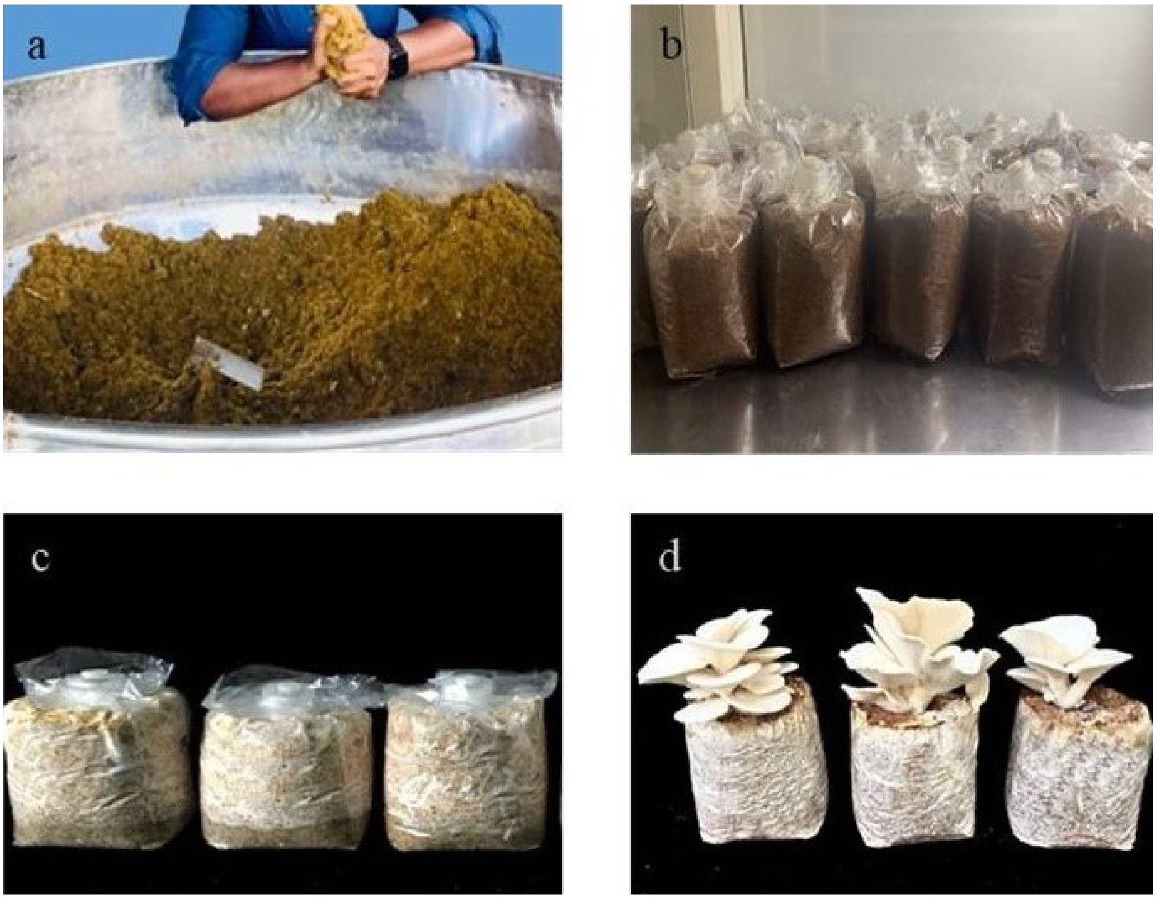

Figure S2. Different stages of oyster mushroom cultivation (*Pleurotus ostreatus*) p969. (**a**) art of substrate preparation (**b**) sterilized substrate ready for injection (**c**) colonized substrate (**d**) first flush for three different grass.
